# Multisensory-inspired modeling and neural correlates for two key binocular interactions

**DOI:** 10.1038/s41598-024-60926-6

**Published:** 2024-05-17

**Authors:** Vincent A. Billock, Kacie Dougherty, Micah J. Kinney, Adam M. Preston, Marc D. Winterbottom

**Affiliations:** 1 Leidos, Inc. at the Naval Aerospace Medical Research Laboratory, NAMRU-D, Wright-Patterson AFB, OH USA; 2 Princeton Neuroscience Institute, Princeton University, Princeton, NJ USA; 3 Naval Air Warfare Center, NAWCAD, Patuxent River, MD USA; 4 Naval Aerospace Medical Research Laboratory, NAMRU-D, Wright-Patterson AFB, OH USA; 5 Air Force Research Laboratory, 711th Human Performance Wing, Wright-Patterson AFB, OH USA

**Keywords:** Sensory processing, Striate cortex

## Abstract

Most binocular vision models assume that the two eyes sum incompletely. However, some facilitatory cortical neurons fire for only one eye, but amplify their firing rates if both eyes are stimulated. These ‘binocular gate’ neurons closely resemble subthreshold multisensory neurons. Binocular amplification for binocular gate neurons follows a power law, with a compressive exponent. Unexpectedly, this rule also applies to facilitatory true binocular neurons; although driven by either eye, binocular neurons are well modeled as gated amplifiers of their strongest monocular response, if both eyes are stimulated. Psychophysical data follows the same power law as the neural data, with a similar exponent; binocular contrast sensitivity can be modeled as a gated amplification of the more sensitive eye. These results resemble gated amplification phenomena in multisensory integration, and other non-driving modulatory interactions that affect sensory processing. Models of incomplete summation seem unnecessary for V1 facilitatory neurons or contrast sensitivity. However, binocular combination of clearly visible monocular stimuli follows Schrödinger’s nonlinear magnitude-weighted average. We find that putatively suppressive binocular neurons closely follow Schrödinger’s equation. Similar suppressive multisensory neurons are well documented but seldom studied. Facilitatory binocular neurons and mildly suppressive binocular neurons are likely neural correlates of binocular sensitivity and binocular appearance respectively.

## Introduction

For eyes, as for many things, two are usually better than one^1–5^. Additive and super-additive binocular enhancements are rare and enhancement usually exceeds that expected for probability summation. For weak stimuli, at the visual threshold, the binocular sensitivity enhancement is usually in the 30–70% range, with 40–50% enhancement most often found^4,6^. For clearly visible stimuli these moderate enhancements are lost and binocular perception behaves more like a nonlinear weighted average of the monocular responses^7^. This study seeks the simplest mathematical rules that closely govern these two behaviors and identifies specific neural mechanisms in macaque visual cortex that behave in accordance with these simple rules; analogous processing in multisensory neurons inspires some of this modeling.

If we begin with the long-standing (circa 1908) universal assumption that binocular enhancement involves summation of monocular responses^8^, then the magnitude of reported enhancements suggest a compressive nonlinearity in the summation (e.g., Eqs. 1–2). In the literature, moderate but significant enhancements in binocular performance are usually referred to as incomplete or partial binocular summation and the term binocular facilitation is used for larger and less common enhancements^8^. However, because our results suggest that the ‘summation’ part of binocular summation might be a misleading misnomer, we will refer – as a theory-neutral term—to ‘binocular enhancement’. In vision research^9,10^ and in multisensory integration^11–13^ subadditive combinations of two sensory channels are often studied using the versatile Minkowski equation,

1 $$Combined \, \;Response = \, (Channel_{1}^{m} + Channel_{2}^{m} + \, \ldots \ldots \, + Channel_{n} )^{1/m} .$$

In binocular vision it takes the form of Eq. (2).

2 $$Binocular\;Combination = \, (Eye_{1}^{m} + Eye_{2}^{m} )^{1/m}$$

At the turn of the twentieth century, Minkowski was a leading authority in geometric theory, and he intended for a slight variation of Eq. (1) to model distances in non-Euclidean spaces^14^. Of course, if *m* = 2, the Euclidean metric applies and Eqs. (1–2) model vector summation. If *m* = 1, the well-known city-block metric applies and summation is additive. As *m* grows, the system becomes more subadditive and for fairly large values can model probability summation.^9^ If inputs are equal the enhancement is just 2^1/m^. In the limit as *m* grows, the subadditive effect of the smaller input becomes negligible compared to the larger input, and Eqs. (1–2) acts like a MAX operator, selecting the largest input. Although not derived from sensory considerations, the Minkowski function’s ease of use has made it the most widespread model for exploring partial summation in general and binocular summation in particular.

**Figure 1:**
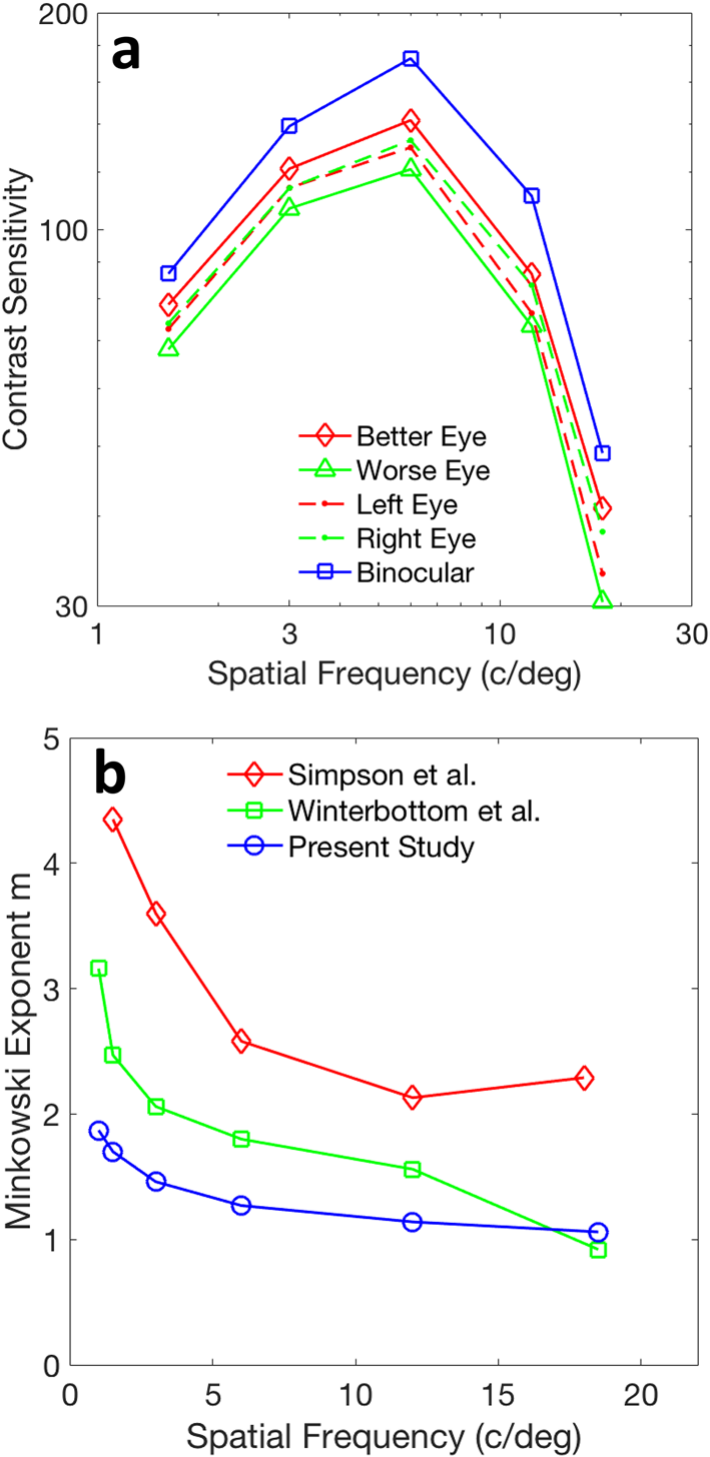
Binocular sensitivity enhancement. (**a**) Psychophysical data^4^ on binocular sensitivity enhancement (average of 45 observers). Most studies show averaged contrast sensitivity as a function of left eye, right eye and binocular contrast sensitivity. Differences in the individual left and right eyes tends to average out, leaving a misleading impression that the left eye and right eye are comparable, but that is not the situation that the binocular brain confronts on a subject-by-subject basis. A truer picture of the binocular task can be obtained by averaging the better eye and worse eye for each subject. (**b**) Applying Minkowski’s formula (Eq. 2) to averaged best monocular/worst monocular/ binocular data, the nonlinearity *m* required to model binocular sensitivity is a function of spatial frequency; high spatial frequencies show more sensitivity enhancement (lower *m*) for binocular stimulation. Similar patterns of spatial frequency dependency are found for three studies (Refs.^4,27^ and the present study).

Attempts to fit binocular enhancement data to Eq. (2) produced *m* values that vary. An early and influential effort by Campbell and Green found an average (of two observers) contrast sensitivity enhancement near 41%,^15^ which was intellectually satisfying because it corresponded closely to arguments from signal detection theory that signal-to-noise ratios for combinations of noisy inputs should grow as the square root of the number of independent channels combined. Likewise, the Minkowski equation would produce an enhancement of 41% for a Minkowski exponent *m* of 2, if the two eyes are equal in sensitivity (they are not quite equal − our direct fit of Campbell and Green’s one published observer’s data to Eq. (2) yields an exponent *m* of 1.85 ± 0.12, which predicts an enhancement factor of 1.45, very close to the true value of 1.44 for this observer). Several subsequent studies used *m* values near 2 to model binocular enhancement under some viewing conditions^16–20^. Related binocular ‘energy’ models, with squaring nonlinearities have also been employed^21^. Other psychophysical studies have found other *m* values, which vary by task^22^. In general, binocular enhancement of brightness in uniform fields is consistent with less summation (larger *m*)^23^, while binocular enhancement of sensitivities for spatial frequency gratings is consistent with more summation (smaller *m*)^4^. The value of *m* often varies with the spatial and temporal frequency content of the stimuli^4,20,23–26^. Some datasets^4,27^ in Fig. 1b illustrate the general finding—moderate-to-high spatial frequencies are more enhanced (have lower* m* values) than lower spatial frequencies.

One original purpose of this study was to understand this spatial frequency dependence. However, because of work in multisensory integration and color vision^28^, we were aware of an alternative model of sensory facilitation which behaves like a gated amplification and has a neural correlate in visual cortex^29–31^. It turns out that this simple multisensory rule^28^—binocularly-gated monocular power law amplification—models binocular contrast sensitivity without taking spatial frequency into account. Indeed, counter-intuitively, there is no need to take the weaker eye into account. Here we show that very similar power laws apply both to psychophysical data and to two classes of neural data in macaque V1, suggesting that neurons as early as V1 could provide the neural correlate of binocular enhancement.

A different rule applies for clearly visible stimuli and a different neural locus must be sought. For suprathreshold stimuli, the binocular response looks more like a nonlinear weighted average of the two eyes, an idea introduced by Erwin Schrödinger in 1926^32^. Let *L* be the left eye response and *R* be the right eye response.

3 $$Binocular\; \, Response = L*\left( {L/\left( {L + R} \right)} \right) \, + R*\left( {R/\left( {L + R} \right)} \right),$$

or equivalently,

4 $$Binocular \, \;Response = \, (L^{{2}} + R^{{2}} ) \, / \, \left( {L + R} \right).$$

The weights are driven by the magnitude of the monocular responses relative to the total response. Schrödinger used this model to understand Fechner’s paradox—the paradoxical increase in binocular brightness that occurs when an observer shuts an artificially impaired eye—and to address binocular color combination. This averaging also reduces brightness differences between binocular-overlap and monocular-only fields-of-view. Very similar equations can be derived from reciprocal monocular suppressive gain-control^33,34^. MacLeod found that Schrödinger’s model—with minor physiologically-inspired modifications—explains much of suprathreshold binocular brightness psychophysics.^7^ Here we identify a class of ‘suppressive’ binocular V1 macaque neurons whose firing rates behave almost exactly like Schrodinger’s binocular equation and thus have the potential to underlie suprathreshold binocular brightness perception. This model may also be applicable to multisensory suppressive neurons. These results are in accord with growing evidence that both driving and modulatory (non-driving) neural interactions are important in sensory processing^35–40^. Here modulatory interactions can account for binocular response at threshold, while driving interactions underlie binocular combination of suprathreshold stimuli.

## Results

### Modeling of neural and psychophysical data on facilitatory binocular interactions

#### Background for modeling binocular gate neurons

Most neurons in V1 are binocularly driven but even putatively monocular V1 neurons show indications that they receive some kind of modulation by the other eye^41–47^. For example Kato et al. studied 112 cortical cells with orientation sensitivity and found that 16 of these neurons (14%) were monocularly-driven^42^. Remarkably 15 out of these 16 putatively monocular neurons showed “clear-cut binocular effects from the silent eye”. These putatively monocular V1 cells are located in Layer 4, but are not LGN afferents; many such cells have oriented receptive fields, including complex and hypercomplex receptive fields and/or motion-direction sensitivity^42,43^. These cells are sometimes called ‘binocular gate neurons’ because the presence of stimulation in the non-driving eye gates the amplification or suppression of the monocular-driving firing rate^42,44–47^. Most studies probed modulatory effects at zero disparity, but several studies also found evidence that an entirely non-driving eye could still produce disparity tuning^48–51^. A recent study in monkey found evidence that the modulatory effects were produced in cortex, likely by feedback from neighboring neurons.^43^ This is consistent with studies that find modulation of Layer 4 neurons from other layers^52,53^. The neural firing rate enhancements resemble the magnitude of enhancement often found for psychophysical binocular contrast sensitivity.

#### Modeling of binocular gate neurons resemble subthresold multisensory neurons and follow the same gated power law monocular amplification

There is a close multisensory analog to the binocular gate neurons. Many multisensory neurons fire for only one kind of stimulation but are strongly modulated by the simultaneous presence of a different sensory stimulation. First discovered in visual–infrared neurons of rattlesnake optic tectum^54^, these cells are now well documented for visual-driven cortical neurons whose firing is modulated (but not driven) by auditory stimulation^29–31^. In the literature these are known as subthreshold multisensory cells or modulated unisensory cells. Billock and Tsou modeled their enhanced neural firing rates with a small neuronal network;^55^ a power law well describes the amplification of the visual signal^28^, with an exponent of 0.87 for Allman et al.’s data.^31^ There are 10 analogous facilitatory binocularly modulated monocular cells in Dougherty et al.’s study of Macaca Radiata.^43^ These binocular gate neurons are driven by only one eye; but if both eyes are simultaneously stimulated, their firing rate increases. Dougherty et al. used the same high contrast spatial frequency gratings as stimuli for both monocular and binocular conditions, with near-optimal predetermined spatial frequencies and a variety of grating orientations for each neuron. We fit the resulting neural firing rates to a power law,

5 $$BinocularFiringRate = a*MonocularFiringRate^{n} .$$

**Figure 2:**
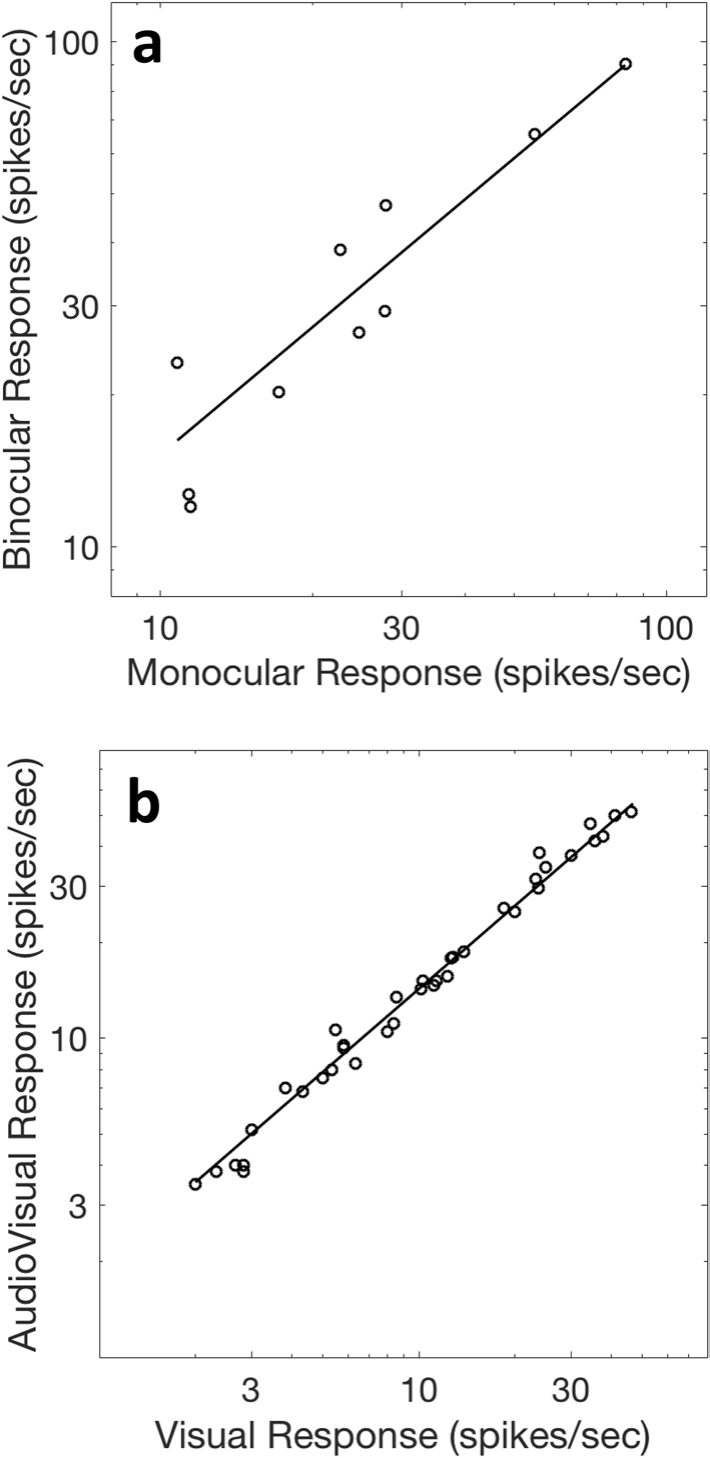
Modeling facilitatory binocular gate neurons with a simple power law model, like the one that models modulatory multisensory cells. (**a**) Dougherty et al.’s 10 facilitatory macaque V1 binocular gate neurons fire for only one eye’s stimulation, but their binocular firing rates are enhanced if the non-driving eye is also stimulated^43^. These neurons are well modeled (r^2^ = 0.93) by a power law with an exponent of 0.84 ± 0.08. (**b**) Comparison to (**a**) of a power law fit^28^ to Allman et al.’s^31^ 37 ‘subthreshold’ multisensory neurons (cat extrastriate visual cortex) that fire for visual stimulation, do not fire for auditory stimulation, but fire harder when both senses are stimulated (*n* = 0.87 ± 0.02; r^2^ = 0.99).

Figure 2a shows the result. The best fit power law has an exponent *n* of 0.84 ± 0.08 (*r*^2^ = 0.93), quite similar to the analogous multisensory (visual modulated by audio) neurons (exponent of 0.87 ± 0.02), with an *r*^2^ value of 0.99 (Fig. 2B). The value of *a* (2.2) is greater than 1—the system is an amplifier; the amplification is reduced nonlinearly by the compressive exponent. Equation 5 amplifies responses to weak/ineffective stimuli relatively better than strong/more-effective stimuli, a result familiar from the sensory integration literature as the Principle of Inverse Effectiveness^56^. This compressive nonlinearity is consistent with the perceptual enhancement of threshold stimuli and the reduced enhancement of stronger stimuli.

#### Modeling true binocular neurons and comparison to the seemingly analogous case of the bimodal multisensory neurons

Just as true binocular neurons can fire to either eye, there are well-studied multisensory ‘bimodal’ neurons, that fire when either sense (say visual and auditory) are stimulated. These multisensory neurons’ spike rates are well fit by the Minkowski model^13^, but are generally less well fit by the power law^28^. This makes sense if neurons are combining responses, however nonlinearly. We would not expect a function of one neural variable to substitute well in what seem like an intrinsically two-variable system—like the two eyes in binocular interactions. So far as we know, this is the first study to fit either power laws or Minkowski functions to the enhanced firing rates of binocular neurons, and consequently the first study to be able to compare a neural parameterization to psychophysical data. For the 55 macaque true facilitatory binocular cells in Dougherty et al.’s’s data, we fit the Minkowski exponent *m* by computing the roots of Eq. (2) (see “Methods”, Eq. 10) and obtained a mean of *m* = 1.77 (an average enhancement of 47%) and a range of 0.38—9.26. Similarly, if we least-squares-fit the whole population of facilitatory binocular neurons to Eq. (2) using a single Minkowski exponent, we obtain an *m* of 1.71 (an average enhancement of 50%) and an *r*^2^ value for the fit of 0.93. These neural values are roughly consistent with typical psychophysical values of the Minkowski exponent (e.g., Fig. 1b) and of the typical reported psychophysical facilitations^4,25^. However, we tried test-fitting the same data to a one-variable power law,

6 $$BinocularFiringRate = a*BestMonocularFiringRate^{n} .$$

**Figure 3:**
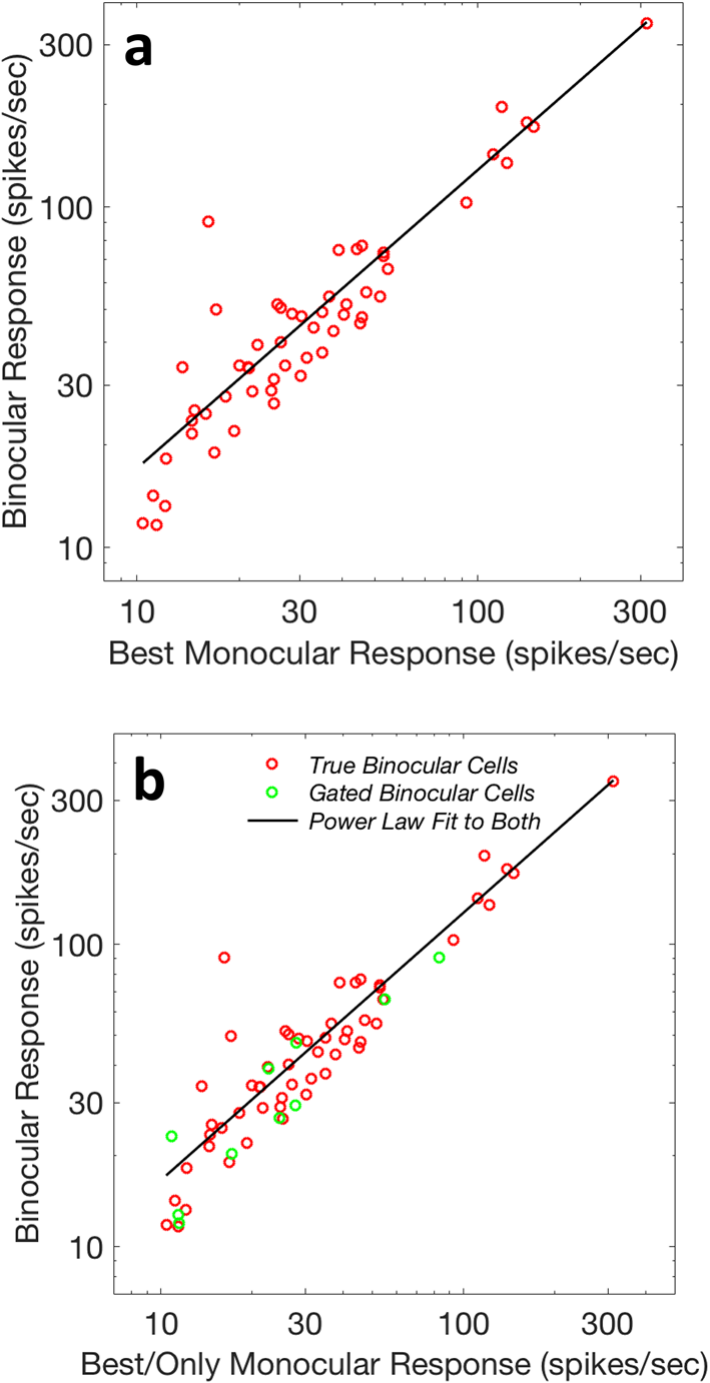
Comparison of power laws for binocular and modulated monocular neurons. (**a**) Modeling of *Dougherty *et al.’s^43^ V1 macaque binocular neurons, which fire for stimulation from either eye but fire harder for binocular stimulation. Surprisingly, a very similar power law model used for the binocular gate neurons applies equally well here (r^2^ = 0.94), with an exponent (*n*) of 0.89 ± 0.03. A Minkowski model does as well (*m* = 1.71 ± 0.13; r^2^ = 0.93). (**b**) The results from the gated binocular and true binocular cells are similar enough that their combined data can be well fit by a power law, with an exponent *n* of 0.90 ± 0.03 and an r^2^ value of 0.93. This combined data cannot be fit with Eq. (2) (because 10 of the neurons lack one of the monocular responses). See Table 1 for model parameterizations.

Surprisingly, this simple one-variable model yields an *r*^2^ value of 0.94 and an exponent of 0.89 (Fig. 3a), similar to the exponent found for the binocular gate neurons (Fig. 2a). Binocular response, which we think of as a function of two variables, can be described at least as well by a function of one variable. When we combine the two neural populations (true binocular and binocular gate neurons) we find a compromise model that does very well for all cortical V1 facilitatory neurons with either kind of binocular input. This fit to all of the facilitatory cells yields a power law exponent of 0.90 and an *r*^2^ value of 0.93 (see Fig. 3b). The Minkowski exponent, for comparison, is not computable for the binocular gate neurons, which lack a second reliable monocular response.

### Modeling of psychophysical binocular enhancement is consistent with neural modeling

The unexpected success of power law amplification for the binocular neurons led us to try the same model in psychophysical binocular enhancement, even though it has always been assumed that binocular enhancement should be a function of both eyes. We modeled two studies that examined binocular enhancement of contrast sensitivity^4,27^ and our own normal control data from an unpublished study of binocular vision with acquired defects in one eye (see “Methods”). Each study used similar spatial frequency ranges, with 5–6 spatial frequencies varying from 1–18.5 c/deg or 1.5–18 c/deg. All three studies were well fit by both Minkowski functions and power laws (see Table 1). In the physical and biological sciences, there is a rule-of-thumb that power law fits should have (at least) about two orders of magnitude range in the dependent and independent variables. By themselves, no one of these psychophysical studies satisfies that criteria. However, there are fairly large differences in monocular and binocular contrast sensitivity in the three studies that are likely driven by stimulus nuances and by differences in the average ages of the subjects in the studies. By combining studies with high and low contrast sensitivities, we can obtain a composite data set that has more range to fit. (This is commonly done in power law studies of physiological size (allometric) scaling relationships, where the individual data points can be different species, ranging say from mice to elephants, or ferns to sequoias.) The psychophysical analog to the neural Eq. (6) is

7 $$BinocularSensitivity = a*BestMonocularSensitivity^{n} .$$

**Table 1:** Power law and Minkowski enhancement model fits to Eqs. (5–7) (*a,n*) or Eq. (2) (*m*)*

Dataset/condition	Number	*a* ± 1se	*n* ± 1se	*m* ± 1se	*r*^2^
Gated Bin Neurons^43^	10	2.20 ± 0.73	0.84 ± 0.08	–	0.931
True Bin Neurons^43^	55	2.14 ± 0.30	0.89 ± 0.03	–	0.935
True Bin Neurons^43^	55	–	–	1.71 ± 0.13	0.929
Combined Bin Neurons^43^	65	2.04 ± 0.26	0.90 ± 0.03	–	0.934
Contrast Sensitivity (new)	9 × 6	1.96 ± 0.40	0.91 ± 0.06	–	0.996
Contrast Sensitivity (new)	9 × 6	–	–	1.60 ± 0.10	0.985
Contrast sensitivity^27^	25 × 6	1.70 ± 0.54	0.90 ± 0.09	–	0.993
Contrast Sensitivity^27^	25 × 6	–	–	2.41 ± 0.20	0.992
Contrast Sensitivity^4^	45 × 5	1.10 ± 0.49	1.02 ± 0.09		0.983
Contrast Sensitivity^4^	45 × 5	–	–	2.86 ± 0.32	0.978
Combined Contrast Sens.	79 × 5/6	1.63 ± 0.23	0.93 ± 0.03		0.990
Combined Contrast Sens.	79 × 5/6	–	–	2.65 ± 0.20	0.986

*Number’ is number of neurons for the first four entries, or number of subjects x number of spatial frequencies for the remaining entries. The Minkowski model is not fit to gated binocular neurons (because the data lack a second input variable). For the Combined Contrast Sensitivity, the number of spatial frequencies (5 or 6) depends on the study.

**Figure 4:**
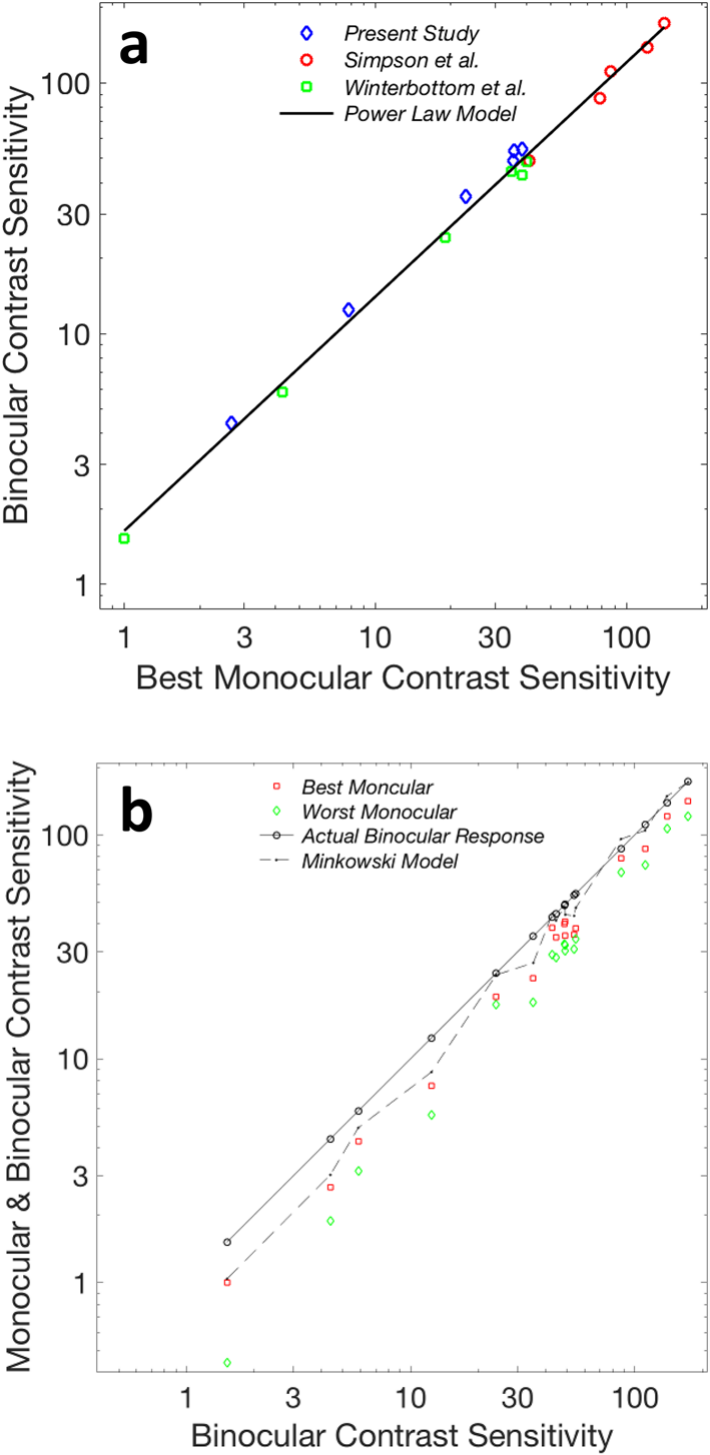
Data from three studies of binocular contrast enhancement. Each data point is the average contrast sensitivity for all observers from one study at one spatial frequency. (**a**) Binocular contrast sensitivity can be well modeled (r^2^ = 0.990) as a simple power law of the more sensitive eye’s contrast sensitivity. The exponent *n* is 0.93 ± 0.03, which resembles the neural data (0.90 ± 0.03, Fig. 3). (**b**) Same data, using both the better and worse monocular responses in the Minkowski equation, to model the binocular response. The special graphing approach, developed for modeling multisensory data^13^ plots monocular and binocular neural responses and modeled binocular response as a function of the binocular neural response. Actual binocular neural data plot (by design) on the 45° axis, monocular inputs plot relative to binocular outputs (illustrating relative monocular contributions to outputs), and model deviations from data stand out. This approach has some advantages for displaying multidimensional data in a readable two-dimensional framework (see “Methods”). The best fit is for a Minkowski exponent *m* of 2.65 ± 0.20, which does not resemble the *m* of 1.71 ± 0.13 found for neural data. The fit is very good (r^2^ = 0.986), with an obvious systematic discrepancy for low-to-moderate contrast sensitivities and a less obvious systematic discrepancy (in the other direction) at higher contrast sensitivities. The use of logarithmic coordinates compresses the positive-going discrepancies at higher contrast sensitivities, relative to low sensitivities.

Figure 4 shows that binocular enhancement behaves like a power law amplification of the more sensitive eye’s contrast sensitivity; the model accounts for 99% of the variance. The exponent is 0.93 ± 0.03, similar to the exponent found for the combined binocular neurons (0.90 ± 0.03). For direct comparison we fit the same data to the Minkowski model for a single value of the Minkowski exponent *m* and got a best fit for *m* = 2.65. The quality of both fits are very good (Table 1), but the residuals for the power law are random while the Minkowski model shows systematic deviations from the data that are most noticeable for low sensitivity conditions (see Fig. 4B), which correspond to the highest spatial frequencies. Since Fig. 1b shows that the best *m* varies with spatial frequency, we could have reduced this discrepancy by using different values of the exponent Minkoswki exponent *m* for different spatial frequencies (and for different studies), but at a cost of adding several more fitting constants. The power law model does not have this problem, in part because of the way that the multiplicative constant and the compressive exponent interact: the combination amplifies weak responses relatively more than strong ones (the famous Principle of Inverse Effectiveness from sensory integration). Because psychophysical contrast sensitivity is weakest at high spatial frequencies, the relative enhancement is stronger at the high frequencies, just as it is in psychophysical data. Another interesting aspect in the modeling is that the Minkowski exponent for binocular neurons (1.71 ± 0.13) does not line up well with the Minkowski exponent for the psychophysical data (2.65 ± 0.20), while the power law exponents for the combined neural (0.90 ± 0.03) and psychophysical data (0.93 ± 0.03) coincide. The power law gated amplification model may be the better model for perceptual and neural data. When comparing psychophysical and neural data by neural subtype, the true binocular neurons are a closer match to the psychophysical data than the gated binocular neurons. However, because the power law exponent for the combined binocular gate neurons and true binocular neurons is so similar to the power law exponent for psychophysical binocular contrast sensitivity, this raises the intriguing prospect that binocular contrast sensitivity draws on the entire pool of enhancing binocular neurons, regardless of subtype.

### Modeling of suppressive binocular neural interactions and relation to suprathreshold binocular vision

#### Background for modeling: suppressive binocular V1 neurons and analogous suppressive multisensory neurons

It has long been known that there are large numbers of binocular neurons whose firing rates lie below their best monocular responses. Most of these neurons are mildly suppressive, with firing rates that lie between the best and worst monocular responses. It has long been unclear what role suppressive binocular neurons play in binocular perception, but their large numbers force us to take them seriously. Similar suppressive neurons are found in many multisensory systems (audiovisual, visual-tactile, audio-tactile and visual–infrared)^54,57^, in many species (cats, ferrets, macaques, rattlesnakes) and their perceptual roles also mostly obscure. Recently here has been suggestions that visual-vestibular neurons, many of which are mildly suppressive, might be mimicking a Bayesian weighted average^58^; and one analysis suggests a particular nonlinear weighted average—invented by Erwin Schrödinger to account for binocular appearance—also models mildly suppressive multisensory neurons^59^. Here we apply Schrödinger’s model and other theoretically interesting models to suppressive binocular neurons.

#### Suppressive binocular V1 neurons seem to implement a specific theoretically meaningful nonlinear weighted binocular average

Schrödinger’s 1926 equation (Eqs. 3–4), a magnitude-weighted nonlinear average of monocular responses^32^, was motivated by Fechner’s 1861 observations on binocular brightness perception.^60^ Very similar equations were later derived from reciprocal suppressive monocular gain control considerations^33,34^. The differences in the new models involve the addition of adjustable parameters that fine-tune the gain control, but we preferred to use the original fully constrained model instead. MacLeod^7^ showed that Schrödinger’s equation could model two studies’ measurements^61,62^ of suprathreshold brightness matching, if the monocular responses were nonlinear functions of luminance differences (contrast). MacLeod used a logarithmic nonlinearity, but the logarithm can closely approximate the Naka-Rushton response functions that are used today. As MacLeod notes, visual neurons with center/surround receptive fields transmit nonlinearly transformed luminance differences to the brain^7^. This suggests that no mathematical extension of Schrodinger’s equation should be necessary to model neural firing rates, because the required transforms to approximate the psychophysical data would already be built into the monocular firing rates. All that remains is to find if there is a class of binocular neurons that follow Schrödinger’s equation in combining the two monocular neural firing rates. MacLeod suggested looking at ‘suppressive’ binocular neurons that fire less for binocular stimulation than for monocular stimulation of the dominant eye. We call this MacLeod’s Criterion.

To be specific, for neurons to be candidates to fit Schrödinger’s equation—or any other weighted average, nonlinear or not—they would have to be only mildly suppressive, in that their binocular firing rate would have to lie between the stronger and weaker monocular firing rates. This is a necessary condition to be a simple weighted average, but it is not a sufficient condition for Schrödinger’s model, since another rule (or none) might govern the weights. For example, a prominent variation on Bayesian sensory reasoning—Maximum Likelihood Estimation (MLE), widely used in sensory integration modeling—would suggest that the weights be inversely proportional to the variances of the monocular firing rates (reliability weighting), rather than the magnitude-based weights of the Schrödinger model^63,64^. For consistency with Dougherty et al.’s data, let *Eye*_*1*_ and *Eye*_2_ be the firing rate of the stronger and weaker responding eyes and *σ*_*1*_^*2*^ and *σ*_*2*_^*2*^ be the associated variances of those firing rates.

8 $$Binocular \, \;Firing\; \, Rate = k_{1} Eye_{1} + k_{2} Eye_{2}$$

where

9 $$k_{1} = \, \left( {{1}/\sigma_{1}^{2} } \right)/\left( {{1}/\sigma_{1}^{2} + { 1}/\sigma_{2}^{2} } \right); k_{2} = \, \left( {{1}/\sigma_{2}^{2} } \right)/\left( {{1}/\sigma_{1}^{2} + { 1}/\sigma_{2}^{2} } \right)$$

**Figure 5:**
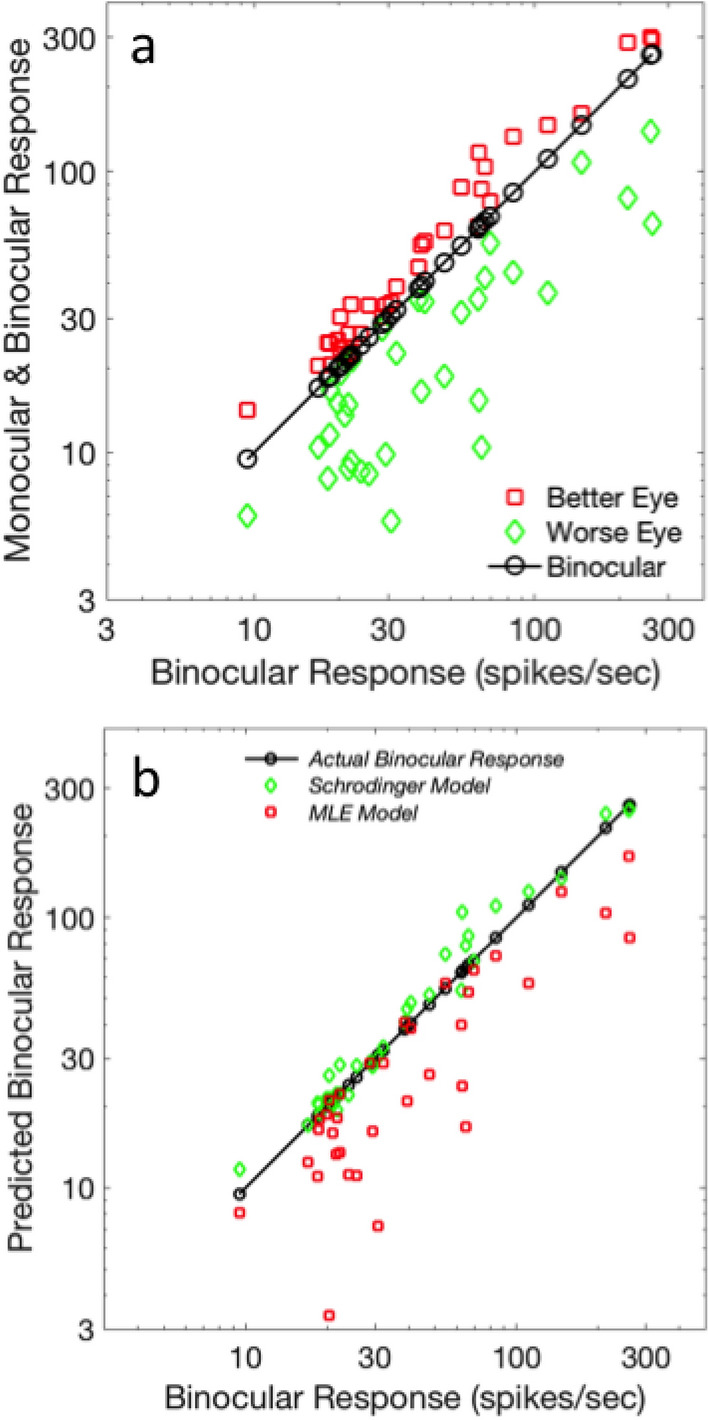
Modeling suppressive binocular neurons. (**a**) Monocular and binocular firing rates for 35 binocular neurons^43^ that meet MacLeod’s criterion for being potential weighted averages of the two eyes. (**b**) Two fully constrained models are plotted with this data using a similar scheme to Fig. 4b; actual neural data plot (by design) on the 45° axis, and model deviations from data stand out. The Schrödinger model works well and has a better fit than the Bayesian MLE model, even if we compensate for the gross underestimation of the MLE model.

Because of the theoretical importance of the Bayesian approach for other neural systems, we test both the Bayesian MLE and Schrödinger models against neural data. Because firing rate variance is strongly correlated to firing rate magnitude, it is obvious that the two models will show somewhat different trends: the Schrödinger model will weight the strongest channel the most (similar to the Minkowski model) while the MLE model will tend to weight the weaker channel more, because it equates reliability with inverse relative variance and variance tends to be correlated with mean firing rate. In addition to their theoretical relevance, both models have the advantage of being perfectly constrained (having no fitting parameters); parameterization is completely determined by firing rate data. Dougherty et al.’s data set^43^ contains 48 suppressive binocular neurons, 35 of which meet MacLeod’s Criterion: these neurons have reliable responses from both eyes and have binocular firing rates that lie between the dominant and non-dominant eye’s responses. We will call these “Between” neurons. Figure 5a shows the ‘Between’ suppressive neurons in Dougherty et al.’s sample, with neural responses to identical high-contrast near-optimal spatial frequency gratings of various orientations, presented parafoveally in a mirror stereoscope. Figure 5b shows the modeling of the ‘Between’ neurons using Bayesian Maximum Likelihood Estimation (Eqs. 8–9) and Schrödinger’s equation (Eq. 3). The MLE model drastically underestimates binocular combination, but with near strict proportionality (a correlation of 0.87), which could allow ‘suppressive’ binocular neurons to serve as substrates for Bayesian-inspired models, if the underestimation is compensated for with a multiplicative constant (in this case 1.61). However, the Schrödinger equation’s performance is much better (overestimating the data by 8%), with strict proportionality (a correlation of 0.98).

## Discussion

### Synopsis

(1) Macaque V1 binocular-facilitatory monocular neurons fire for stimulation in one eye, but not the other, and have enhanced responses when both eyes are stimulated. The activity in the non-driving eye gates the power law amplification of the response to stimulation in the eye that drives the neuron’s firing rate. This result has a sensory precedent—visual cortical neurons amplified by auditory inputs^31^; these modulated unisensory neurons have very similar power law exponents to the modulated monocular neurons^28^. (2) For true binocular neurons, which fire for stimulation of either eye, binocular facilitation can be predicted as a power law amplification of the stronger monocular response. The variance accounted for (94%) suggests that there is little need for a more complicated model that would take the weaker eye into account. This is surprising because power law models do not fully account for facilitation in multisensory bimodal cells (a multisensory analog of binocular neurons), necessitating taking the second variable into account for bimodal neurons^28^. The Minkowski model does almost as well for the true binocular neurons, but can’t be extended to cover binocular gate neurons, while the power law can. (3) The slightly compressive power law exponents means that weakly stimulated neurons will be relatively more binocularly amplified than strong ones. This predicts less binocular enhancement for stronger (clearly visible) stimuli. In sensory integration this behavior is known as the Principle of Inverse Effectiveness. (4) Psychophysical data follows the neural trend: binocular sensitivity is well modeled as a power law of the greater monocular sensitivity. The exponent for psychophysical data is similar to the exponent for binocular neurons and the *r*^2^ value of 0.99 suggests that more complicated models—taking the weaker eye into account—would be of limited utility. The similarity of power law exponents for V1 excitatory neurons and for psychophysics suggests that these cortical neurons could be the correlates of psychophysical binocular enhancement at threshold. (5) The exponent for the combined neural populations (0.90 ± 0.03) closely resembles the exponent for the psychophysical data (0.93 ± 0.03). This result suggests that both gated binocular neurons and true binocular neurons could be contributing to psychophysical binocular facilitation. (6) These results are consistent with Sherman and Guillery’s driver/modulator framework^35^, since both neural data sets and the three psychophysical studies are all consistent with input from the weaker (or non-driving) eye gating the amplification of the stronger (or driving eye); the actual strength of the weaker eye’s signal is not noticeably a factor in the resulting amplification. It is possible to build simple similar-behaving neuronal networks^55^, whose firing rates closely approximate power law amplification^28^. (7) So far as we know, these are the first models of binocular enhancement that do not involve nonlinear summation of the two eyes and this is the first quantitative study to directly compare binocular enhancement interactions in neurons and perception. (8) MacLeod found that Schrödinger’s binocular equation, with extensions for neural attributes, models suprathreshold binocular brightness^7^. Here we showed that Schrödinger’s equation is a tight match to the spike rate behavior of mildly suppressive (‘Between’) binocular neurons which suppress binocular firing rate relative to dominant eye stimulation firing rate: both are simple nonlinear-weighted averages of monocular spike rates. (9) It follows that facilitatory modulated (“gated”) binocular neurons and facilitatory binocular-driven neurons are both potential neural correlates of psychophysical threshold binocular enhancements, and that mildly suppressive binocular “Between” neurons are a potential neural correlate of supratheshold binocular brightness perception.

### An experimental prediction for binocular enhancement of contrast sensitivity

The data for both binocular-driven and binocular-modulated neurons, and for binocular contrast sensitivity, is consistent with gated power law amplification of the more sensitive eye’s responses. The activity driven by the less responsive eye gates the amplification of the monocular signal but does not otherwise participate in the calculation, so the actual magnitude of the weaker eye’s response is not required to model the data. It seems likely that there is a threshold level of activation by the weaker eye’s response that triggers the gated amplification, but the threshold can’t be high in binocular gate neurons, since the binocular amplification obtains even for these neurons that don’t reliably generate spikes for the weaker eye’s stimulation (e.g., Fig. 2a). Similarly, we have preliminary unpublished psychophysical evidence that applying a moderate amount of blur to either eye does not abolish binocular amplification of the unfiltered eye. Although some studies show little-to-no binocular enhancement in amblyopes, Baker et al. found that they could get normal binocular summation in amblyopes by compensating for the weaker monocular response with a stronger stimulus^65^. Similarly Schneck et al. found little-to-no binocular enhancement in elderly subjects when there was a strong difference in stimulation of the two eyes^66^. It would be interesting to measure the amount of blur or the reduction of contrast applied to one eye that suffices to abolish the enhancement, and determine whether this threshold is fixed or labile.

### An experimental prediction for mildly suppressive “between” neurons

The evidence above suggests that mildly suppressive “Between” cells are a likely locus of weighted averaging in suprathreshold binocular perception and are the locus of the calculations embodied in the Schrödinger model. For this to be true the between-status of ‘Between’ cells has to be reliable—the binocular response should almost always lie between the monocular responses. This needs to be tested experimentally with a range of stimuli to verify that cells that test as weighted averagers for one stimulus pair also test as weighted averagers for other stimuli. An alternative would be for the visual system to pool all of the suppressive cells to compute averages, but this result would not be as clean as using a stable reliable ‘Between’ cell population.

### The modulator/driver distinction

Sherman and Guillery’s^35^ modulator/driver distinction is an influential framework for understanding neural interactions^36–40^. Although it has long been apparent that some neurons do not cause their postsynaptic neurons to spike, they can still modulate the target neuron’s output when it is already firing. Here we have discussed both modulated unisensory neurons ^28–31,54,55^ and modulated monocular neurons^42–47^ as examples where one of the neural inputs is driving and the other is modulatory. Modulator neurons / gated psychophysical amplifications are also found in several other sensory contexts, including color vision, attention and especially in thalamic processing^28,35–40^. This modulation can modify sensitivity tuning of cortical neurons. Amplification with an expansive exponent results in sharper tuning, while a compressive exponent (found both here and in Ref.^28^) broadens tuning by amplifying weak responses more than strong ones.

### Occam’s Razor and binocular integration at threshold

Some aspects of binocular integration definitely combine information from both eyes—stereopsis (and binocular luster) would not be possible otherwise. However, the present results show an interesting disconnect between binocular enhancement and stereopsis: binocular enhancement is consistent with binocularly gated amplification of monocular signals. Of course this had to be the case for the binocularly-modulated monocular (gated binocular) neurons, since they fire for only one eye and are modulated by activation of the other eye. But this did not have to be true for explicitly binocular neurons or for psychophysical data. Indeed we would have predicted otherwise. However, all three classes of data are well modeled by a simple power law amplification of monocular signals, with similar exponents. Conversely, it is never surprising that a Minkowski function can fit two monocular responses to a binocular response because if the binocular response is greater than either monocular response, there exists a Minkowski exponent that satisfies the equation, no matter how arbitrary the monocular values are. Although the Minkowski exponent *m* fit to contrast sensitivity data varies somewhat with spatial frequency (Fig. 1b), a compromise exponent does a surprisingly good job of fitting entire datasets, with fits that are almost as good as those for the power law. Similarly, the Minkowski model does almost as well for the binocular-driven neurons, but can’t be extended to cover binocular gate neurons, the way that the power law can. Moreover, the estimated power law exponents for neural and psychophysical data are more consistent with each other than the Minkowski exponents for the same data (Table 1). Considering just psychophysical data (where we have multiple data sets), the power law exponents are more consistent between data sets than the Minkowski exponents. Although some kind of nonlinear summation can’t be ruled out for some kinds of binocular enhancement, Occam’s Razor would seem to apply here: if gated amplification, a function of one variable (dominant eye), can account for 99% of the experimental variance in psychophysical data and 93–94% of the variance in both kinds of binocular neural firing rates, then the role of the other variable (non-dominant/non-driving eye) is (in those contexts) negligible. The consequences of adopting a one-variable model are: (a) rethinking the venerable notion that binocular enhancement is a form of summation and (b) taking seriously the notion that modulatory interactions can modify sensory performance. It is intriguing to consider the disconnect between a monocularly-driven binocular enhancement model and the phenomenon of stereopsis. A partial reconciliation is inherent in a recent model of stereopsis that utilizes monocularly-dominated inputs^67^. It is interesting that a recent study of pheromone interaction in insects—a phenomenon that was previously modeled using both pheromones—has been shown to be well-modeled using one pheromone^68^; the data obey the Billock and Havig^28^ gated amplification model (used here for binocular enhancement) , but with a more compressive exponent. It may be that other systems that employ two-variable models, like binaural and bivestibular interactions, might be profitably analyzed with a single variable model.

### Comparing power laws for neural and psychophysical data

Power laws frequently arise in psychophysics and are often used in neuroscience. It is also not uncommon for power laws for particular classes of neurons to have similar exponents as psychophysical data. Here we found that visual neurons that amplify their responses when the stimuli are binocular obey a power law with an exponent of 0.90 ± 0.03. Similarly, the power law for binocular contrast sensitivity amplification has a power law of with an exponent of 0.93 ± -0.03. Similar coincidences have arisen in many other comparisons of neural and psychophysical behavior. The most relevant one for our purposes arises from audio-visual amplifications. For example Stein et al.^69^ published data on audio effects on perceived brightness of various dim lights. Billock and Havig^28^ fit the audio-amplified data as a function of the visual-alone data to a power law with an exponent of 0.82. For comparison they fit data on visual neurons that amplify their firing rates when audio signals are present and got a power law of 0.87. Other, somewhat related examples can be found in the Stevens’ law literature (Billock and Havig’s modeling^28^ can be thought of as a second-order Steven’s law, arising as a ratio of Stevens’ laws for amplified and unamplified data respectively). Stevens reviews several such psychophysical and neural coincidences^69,70^. Two particularly interesting cases arise from consideration of the brightness of lights measured for conditions with and without physical contrast: (a) Several investigators, including Stevens find that apparent brightness of targets on a different background grows as the cube root of the intensity of the target^69,70^. Similarly Stevens notes that reaction time drops and neural responses in both peripheral neurons and central measures (evoked potentials) grow as the cube root of intensity for contrasting targets. (b) A different power law for non-contrasting targets governs pupil contraction (an objective measure of neurally controlled subjective brightness intensity); the exponent is 0.22^72^, while the power law for brightness perception in a Ganzfeld (a uniform target filling the visual field) is 0.24^72,73^. If taken seriously, matches between psychophysical and neural response functions have interesting theoretical consequences^74–77^. As Johnson et al.^75^ note, if both neural and psychophysical data have response functions of the same form, they can be expressed as a linear function of one another, i.e.,

10 $$Psychophysical = k*Neural.$$

In the case of neural and psychophysical power laws with the same exponent, any differences in the fitted proportionality constants of the power laws would be captured in the value of ‘*k*’. To our knowledge no one has designed experiments to probe this exact aspect of the modeling, but Johnson et al. have done the most rigorous work (on the linear relationship between texture perception and its best neural correlate)^75^. More interesting, as Werner and Montcastle^77^ point out, if both the psychophysical and neural functions have the same mathematical form (and if this is not just a coincidence), then any subsequent non-measured neural processing must not cumulatively distort the measured functional response, beyond changing the value of ‘*k*’ in Eq. (10).

### Similarity between multisensory and binocular interactions

Mildly suppressive binocular neurons—which are well fit by Schrodinger’s nonlinear magnitude weighted average—behave much like most multisensory suppressive neurons. That is, multisensory neurons deemed suppressive are categorized as suppressive because their response to multisensory stimulation is lower than their best response to individual unisensory stimuli. But most such neurons are not strongly suppressive: their multisensory response lies between the best and worse unisensory responses. As such they are good candidates for being modeled by a weighted average, but which one? A preliminary report from our laboratory found that in each of three tested cases (visual-tactile, audio-tactile and audio-visual), multisensory Between-neurons were well fit by the Schrodinger model and that reliability weighting did not do quite as well^59^. For binocular facilitation we found two candidate neural classes: true binocular neurons and gated binocular neurons. True binocular facilitatory neurons fire for stimulation in either eye, but fire harder for binocular stimulation than for either eye stimulated alone. Binocular gate neurons fire for stimulation in one eye, but not the other, but still fire harder when both eyes are stimulated. On their face, true binocular neurons resemble multisensory bimodal cells and binocular gate neurons resemble what various multisensory labs have called subthreshold multisensory cells or modulated unisensory cells. Most of the work on these units has been done in cat extrastriate visual cortex and find neurons that fire for visual stimuli, don’t fire for auditory stimulation, but fire harder when an audio stimulus accompanies a visual stimulus. Our previous modeling (Fig. 2b) of the subthreshold audio-visual neurons found that they were well fit by a power law with an exponent of 0.87 ± 0.02. This closely resembles the exponent of 0.84 ± 0.08 which we found for the gated binocular neurons. Since true binocular cells behave analogously to bimodal multisensory cells and we had found previously that the Minkowski incomplete summation rule works well for bimodal sensory cells, we expected the Minkowski equation to be a good model for true binocular neurons, and it was. It was unexpected however that the power law would provide such a good model for true binocular neurons.

### Limitations of the study and limitations of power law models

Our psychophysical data was from studies of binocular contrast sensitivity. We are aware of (but do not presently have data to model) studies of color and contrast interactions in binocular vision. We do not know if these studies could be well modeled at threshold by the power law. Similarly, we make no argument that the true form of the binocular sensitivity is a power law, only that it provides the simplest model with an excellent and consistent fit to both psychophysical and neural data. For example, our neural amplifier model with Hodgkin–Huxley dynamics^55^ results in a function that closely resembles a power law, with small periodic excursions around the power law fit (see Fig. 4c of Ref.^28^). Establishing that a function is actually a power law (and not another member of the family of power-law-like functions) requires more data and a larger response range than is at our disposal. We use the power law here to connect the data to similar results in sensory integration and color vision^28,78^ and to show that no knowledge of the weaker eye’s response (other than it is active) is necessary to model these neural and psychophysical data. Related findings in sensory integration and color vision interactions^28^ suggest that modulatory neurons may play roles in perception in parallel with the role played by the better-studied driver neurons. It would be particularly interesting to probe these neural subtypes roles in binocular enhancement using experiments that compare neurometric and psychometric functions in the same behaving animals. Similar caveats apply to the Schrödinger model for suprathreshold perception. In any case, we have identified two remarkably simple rules followed by binocular interactions in both perception and in some neural populations.

## Methods

### Experimental methods

All of the neural^41^ and psychophysical data^4,27^ modeled in this paper have already been published (several by our combined laboratories) except for an excerpt from our unpublished (Kinney & Billock) pilot data that was included in the modeling in Figs. 1a and 4. These pilot data motivated a closer look at the other more extensive datasets analyzed here, and were taken from a larger unpublished project that examined the clinical effects of monocular disease in binocular vision, but was cut short by the COVID pandemic. The pilot data used in this paper were monocular and binocular contrast sensitivities measured in nine normal control observers. Because the methods were identical to those used in another published study by our group^27^, and the apparatus was the apparatus used in that study, we briefly summarize here the more detailed methods found in Ref. [27]. Briefly, the nine subjects, who were in their twenties and thirties, all had normal or corrected-to-normal vision. Contrast sensitivity was measured using a filtered optotype approach with the ‘quick CSF’ methodology of Ref. [79]. The commercially available (Adaptive Sensory Technology) apparatus is tablet-based and uses a variety of spatially filtered optotypes^80^. The variation that we employed was a prototype created by AST for the US Air Force School of Aerospace Medicine that employs spatially bandpass filtered Landolt Cs presented on a NEC Multisync P463 flat panel display^27^. Resolution on the 46″ display was 1920 × 1080, with a contrast ratio of 4000:1. Images were viewed at 4 m and were scaled to have centroid spatial frequencies of 1, 1.5, 3, 6, 12, and 18.5 c/deg. An interlaced presentation, four-alternative forced choice, Bayesian adaptive procedure was used to estimate thresholds for each stimulus^79^. The experiment was run for both eyes separately and for binocular combination. Five of the nine observers returned for a second session before their service was cut short by the COVID pandemic, and data from the first and second sessions were averaged. The study was approved by the Institutional Review Board at the Naval Medical Research Unit—Dayton and complied with the Declaration of Helsinki. Informed consent was obtained from all subjects. The subjects performed all of the experiments in a refraction lane and viewed stimuli through a clinical phoropter (NIDEK RT5100, Aichi, Japan). The phoropter was used to refract subjects to their optical correction, to add prism and polarization to align and prepare monocular images for binocular fusion (to study sensory eye dominance and binocular rivalry for each spatial frequency) and for some experiments to degrade one monocular image relative to another. The data on rivalry, dominance and degraded images are not treated here and will be employed in a different venue.

### Exclusion of non-facilitatory conditions/subjects and related calculations

Facilitatory neurons are defined as neurons that show binocular spike rate enhancement on a majority of trials, even if a few trials don’t show enhancement. When comparing neural and psychophysical behaviors, it would be most comparable to use subjects who show binocular enhancement at a majority of spatial frequencies. Most subjects show binocular enhancement and most show enhancement at each spatial frequency. For example, in Simpson et al.’s dataset,^4^ only one of 51 subjects showed no binocular enhancement and only five other subjects failed to show enhancement at a majority of spatial frequencies tested. Similarly, in the Winterbottom et al*.* dataset,^27^ we excluded five subjects who showed no enhancement at most spatial frequencies; several of these subjects had notations in their records about amblyopia and other binocular problems. The remaining subjects had their monocular and binocular data averaged and the averaged best eye sensitivity and binocular sensitivity was used to compute the Eq. (6) power law in Fig. 4a. All fits, both for power laws and Minkowski’s equation, were performed in CurveExpertPro 2.5 (Hyams Development, Chattanooga, TN).

The Minkowski equation (Eq. 2) has no valid solution unless binocular sensitivity exceeds sensitivity for each monocular condition; the Minkowski equation was derived from Pythagorean-like distances in non-Euclidean geometries and in no sensible geometry are combined dimensional distances less than the largest component distance. If this condition holds, then one way to estimate the Minkowski exponent *m* for each spatial frequency is to calculate the Minkowski exponents for every observer at that spatial frequency and average the exponents. This can be done by root-finding, e.g., by solving for the values of *m* that satisfy Eq. (10).

11 $$BinocularSen{-}(BestEyeSen^{m} + WorstEyeSen^{m} )^{1/m} = \, 0$$

The root-finding was via the Forsythe algorithm (a hybrid algorithm that combines bisection, secant and inverse quadratic interpolating methods)^81^, as implemented in MATLAB.

One potential limitation: if binocular sensitivity is only barely above one or both of the monocular sensitivities, then Eq. (11) returns extreme values. For some subjects who had binocular enhancement at a majority of spatial frequencies, we must also exclude subjects who did not show enhancement at a particular spatial frequency for that spatial frequency calculation. Alternately, one could use a nonlinear least-squares fit to all the individual data at each spatial frequency^4^. This method was used to compute the exponent for the fit shown in Fig. 4b. A third way to compute* m* is to average the sensitivity data at each spatial frequency and then compute *m* from the averaged data using root finding. The three methods yield similar results; the results of the third method are plotted in Fig. 1b to illustrate the spatial frequency dependence of the Minkowski exponent.

### Plotting binocular three dimensional input/output data in two dimensional graphs

For Figs. 4b and 5 we used a new method for plotting three dimensional input–output data in two dimensions that was originally created to plot multisensory data^13^. The standard practice in multisensory science is to plot multisensory combination data as a function of the strongest mono-sensory response. This practice was invented to illustrate multisensory enhancement but does not aid understanding of how inputs combine. The use of three-dimensional prospective plots is possible but it is difficult to examine model fits in such a plot and two-dimensional contour plots can be difficult to interpret. The existence of a solution to this problem in the multisensory domain makes the technique useful for the plots of binocular relationships. Both binocular and monocular responses are plotted as a function of the binocular response. This places the binocular data on a straight 45 degree line, in any monotonic coordinate system. The two monocular responses are placed relative to the binocular response so the reader can instantly see the potential relative contributions of the two inputs to the binocular outputs. Model discrepancies pop out as deviations from the straight line. Here, the best-fit Minkowski binocular model was then simulated from the monocular responses and the simulated binocular responses were overlaid on the graph. An alternative would have been to plot the two monocular responses, the binocular response, and the modeled response as functions of some fifth variable, but in this case a fifth variable was not available and the option of creating a dummy variable seemed unnecessary and excessively convoluted.

## Data Availability

Data and code original to this paper will be shared by the lead contact (V.A. Billock, vincent.billock.ctr@us.af.mil) on request.
